# The understanding, acceptability, and relevance of personalised multidimensional physical activity feedback among urban adults: evidence from a qualitative feasibility study in Sri Lanka

**DOI:** 10.1186/s12889-021-10774-0

**Published:** 2021-04-13

**Authors:** Carukshi Arambepola, Madhawa Perera, Fiona Gillison, Oliver Peacock, Dylan Thompson

**Affiliations:** 1 Department of Community Medicine, Faculty of Medicine, University of Colombo, Colombo, Sri Lanka; 2 Department of Physiology, Faculty of Medicine, University of Peradeniya, Peradeniya, Sri Lanka; 3 Department for Health, University of Bath, Bath, UK

**Keywords:** Multidimensional personalised physical activity profile, Activity monitor, Feasibility, Sri Lanka

## Abstract

**Background:**

Wearable technologies are being used to provide personalised feedback across multiple physical activity dimensions in countries such as the UK, but their feasibility has not been tested in South Asia, where physical inactivity is increasing. This study assessed the understanding, acceptability, and relevance of personalised multidimensional physical activity feedback in urban dwellers in Colombo, Sri Lanka.

**Methods:**

A qualitative feasibility study was conducted among 35 adults to assess a community-based approach to provide multidimensional physical activity feedback. Healthy adults, adults at risk of non-communicable diseases and community-based primary healthcare professionals wore a physical activity monitor for 7 days and were then guided through their personalised multidimensional physical activity feedback. One-to-one interviews were conducted, transcribed verbatim and analysed using framework analysis.

**Results:**

Four themes were generated: understanding of personalised physical activity feedback, perceived novelty of the feedback, motivation, and consideration of the multidimensional nature of physical activity. A majority of participants required guidance initially to understand the feedback, following which most were quickly able to interpret the data shown, and were willing to use the feedback as a basis for identifying goals to improve physical activity. Participants perceived the feedback and its delivery as novel because it provided new knowledge about physical activity guidelines and awareness on their own behaviour through graphics. Comparisons of personal performance against recommended physical activity levels and information on sedentary time were the most commonly motivating aspects of the feedback, prompting talk about behaviour change. All three groups showed poor planning on goal achievement, with some noticeable differences between those with and without health risk of non-communicable diseases. Following the feedback, most participants understood that physical activity is composed of several dimensions, while around half could recognise more suitable options to change behaviour. Of the physical activity dimensions, calorie burn received more attention than others.

**Conclusions:**

Multidimensional physical activity feedback was considered understandable and acceptable and has the potential to support behaviour change among urban Sri Lankans with or without identified health risk. These findings highlight the feasibility of this technology-enabled approach as a personalised intervention to improve knowledge and motivation for physical activity behaviour.

**Supplementary Information:**

The online version contains supplementary material available at 10.1186/s12889-021-10774-0.

## Background

It is well-known that physical activity (PA) promotion is complex and that translating PA recommendations into action is challenging [1, 2], in part because of the difficulty associated with the clear provision of feedback on the type, duration and frequency of activities carried out [3]. In this regard, wearable devices enable individuals to self-monitor and manage their PA and self-monitoring empowers adults to engage in more feasible and effective PA [4, 5]. With improvements in accuracy and precision [6], wearable devices represent a scalable and low-cost strategy to potentially help individuals change their behaviour [4, 7].

It is increasingly recognised that PA is a highly heterogeneous and complex behaviour with multiple dimensions, which are known to be independently important for health [8–11]. However, previous research studies that use self-monitoring often focus only on feedback of a single PA metric (e.g. step counts). Such unidimensional feedback may not be interpreted as relevant or informative, in terms of enabling the recipient to identify how different aspects of their behaviour contribute to overall PA levels.

Recently, novel device-based approaches have been used to capture different physiologically important dimensions of PA by generating personalised multidimensional PA ‘profiles’ [12]. These profiles have the potential to overcome the heterogenic nature of PA and enable individuals to form a more holistic view of their habitual PA. Feedback of visual multidimensional PA profiles has been shown to be informative and motivating in Europeans [11, 13, 14]. This approach is now being used in the UK National Health Service (NHS), but it has not yet been tested in developing countries, where the context of communication around PA may be different. For example, it is well established that people generally prefer visual and meaningful images for health communication [15, 16], but it is not clear whether the same visualisations are useful across populations and settings. Therefore, it is important to establish local understanding, acceptability, and relevance whenever health related visual feedback is introduced into a country [16, 17].

Physical inactivity is a rapidly expanding public health problem in South Asia, including in Sri Lanka where it has dramatically increased [18, 19]. Despite the estimated contribution of physical inactivity to non-communicable diseases (NCD) and all-cause mortality [20], there are major barriers to promotion of PA in Sri Lanka. Among these, over-estimation of PA level [21], is a crucial barrier, which is also a well-proven phenomenon worldwide, especially among inactive adults [22]. Against this backdrop, the aim of this study was to explore the acceptability and perceived relevance of providing personalised visual multidimensional PA feedback via a web-based platform to promote the understanding of personalised PA profiles among Sri Lankan adults.

## Methods

### Participants and recruitment

A qualitative feasibility study was conducted in Colombo District, Sri Lanka in a geographically defined administrative area, which represented ‘typical’ urban dwellers of Sri Lanka. Three groups of participants aged 20–60 who had been living or working for a minimum period of 1 year in one of the health administrative units (medical officer of health areas) in Sri Lanka were recruited: (1) Apparently healthy adults, (2) Adults at risk of chronic NCDs and (3) community-based primary healthcare professionals (e.g. public health midwives, nursing sisters and inspectors). The latter group was included in the study to capture the views of primary healthcare professionals who work in urban settings, as it is important to gauge their understanding on the feedback, if these technologies are going to be used in such settings. In the study, those at risk of NCDs were identified by the presence of two or more metabolic risk factors for NCDs (hypertension, dyslipidaemia, hyperglycaemia, obesity), while apparently healthy persons were identified by the absence of these risk factors. The diagnosis of hypertension, diabetes and dyslipidaemia was confirmed by documental evidence, based on clinic records, drug prescriptions or laboratory testing; and obesity by measuring Body Mass Index. Those having physical disabilities (e.g. limb deformities, blindness), stroke, heart disease, acute illness or any contra-indication for being active (e.g. medical reasons) or for the wearable device (e.g. allergies to metal or silicon strap), and pregnant women were excluded from all three groups.

The sampling was done purposively with the assistance of the area public health midwives, in order to include both working and non-working males and females of multi-ethnic and varying socio-economic status from streets that were typically classified by the local community as ‘affluent’, ‘middle-income’ and ‘low-income’. The sample was recruited from individual households, workplaces, primary healthcare units and fitness centres in the defined geographical area. The sample size was determined by the point at which no more new information was generated by the participants in each group.

### Procedure

**Fig. 1:**
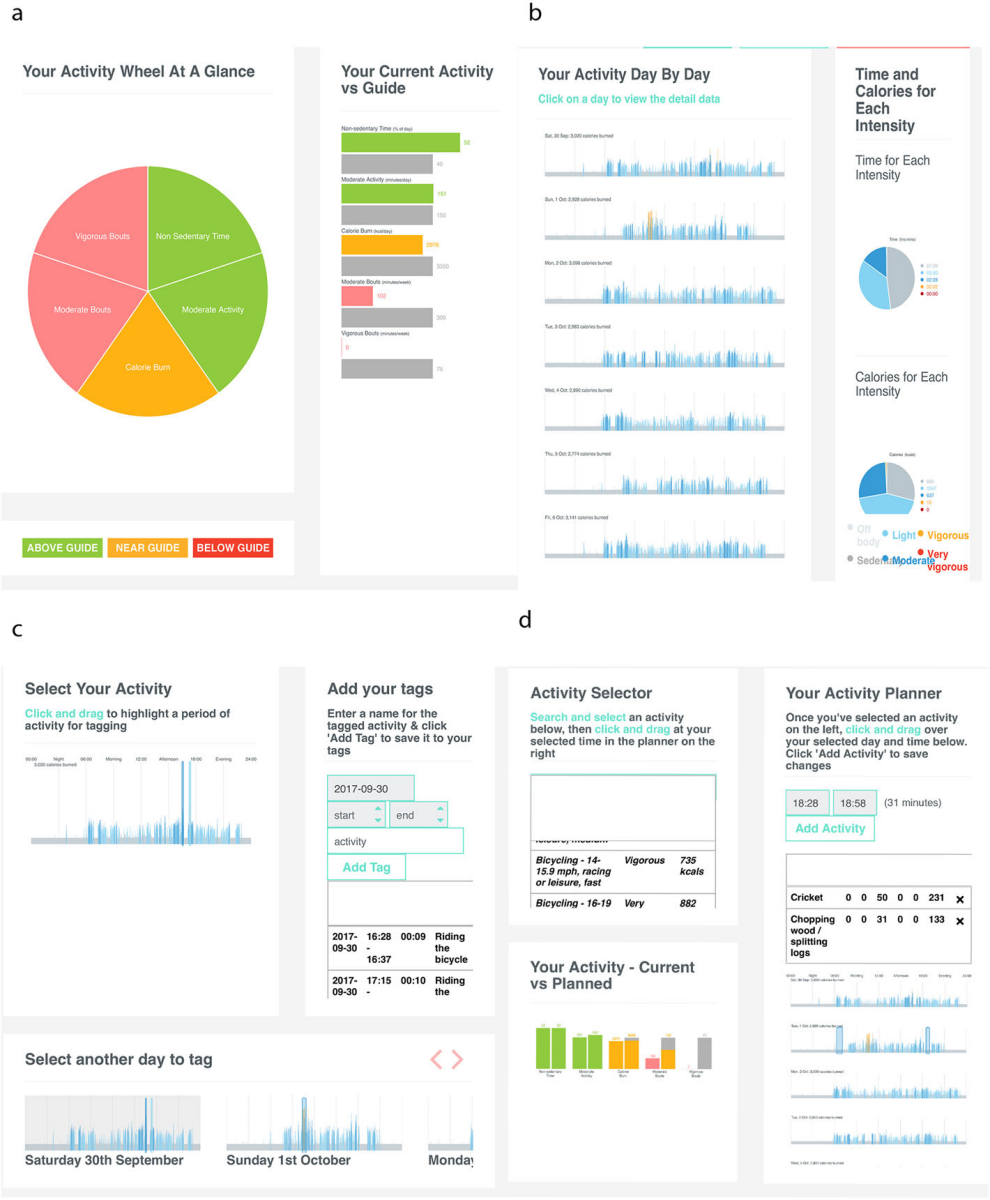
**a**: Section 1 – Health Profile (types of activities of the past week shown in an activity wheel and against the guide). **b**: Section 2 – Day by day activity (activities of the past week shown in day by day graphs and time & calories for each intensity). **c**: Section 3 – Tagging your activity (select activities carried out during past week and add tags). **d**: Section 4 – Plan to improve (activity selector, activity planner and current vs. planned activities)

Ethics approval for the study was granted by Ethics Review Committee of the Faculty of Medicine, University of Colombo, Sri Lanka. Following informed written consent, each participant was provided with a PA monitor, including clear written and verbal instructions for use. The device was a FitNLife activity monitor (Fit.Life Inc., South Korea) which is a lightweight commercially available accelerometer worn on the wrist. Participants were asked to wear the PA monitor for seven consecutive days (including when sleeping, but not during water-based activities) and advised not to change their usual activity pattern. The device did not display information or feedback to the user. To minimize non-compliance (wear time) and to resolve any technical issues, daily reminders were given via telephone and text messages. On the eighth day, the investigator scheduled a meeting with each participant to provide visual feedback of his/her PA profile using a customised web-platform (https://kiactiv.com). This platform automatically generated multi-faceted visual feedback as previously examined in a UK population [13] based on five dimensions of their behaviour: (1) overall energy expenditure, (2) time engaged in moderate-vigorous physical activities (MVPA) on a minute-by-minute basis, (3) time engaged in MVPA accumulated in bouts of at least 10 min, (4) time engaged in VPA accumulated in bouts of at least 10 min and, (5) non-sedentary time (an example of such profiling is given in Fig. 1). A semi-structured in-depth interview was conducted in the local language (Sinhala), while guiding each participant through the visual feedback. The visual feedback consisted of four main sections (health profile, activity, tagging and planning), where a brief explanation about each section was given to the participants at the beginning of the section. If the participants failed to understand the visual feedback after the initial explanation, they were provided some extra support by explaining the relevant graphical components which they could not understand. At this stage they were explained the selected graphics taking examples from their own PA profile. The interviews were recorded using a digital audio-recorder and later transcribed in Sinhala and translated to English. All the transcriptions were anonymised to secure the confidentiality of the respondents.

### Interview topic guide

The topic guide was based on a previous similar study with a UK population [13], which was culturally adapted for urban Sri Lankan participants through consultation with seven lay persons representing the target group, and four experts in the fields of exercise and sports science, community medicine, education and psychology.

Themes initially included in the guide were: interpretation of the PA profile (perception and understanding of the personalized profile linked to information graphics); acceptability of the visual feedback; and the relevance of PA profiles to motivate participants for enhancing their level of PA (interviewer topic guide given as Additional file 1).

### Data analysis

Qualitative data were analysed using framework analysis. A period of familiarisation with the transcripts by the investigator was followed by a process of coding whereby initial themes directed by the interview topic guide, unexpected emergent themes and periodic concepts raised by the interviewees were identified. The appropriateness of initial themes, derived from a subset of the transcripts, was confirmed by two investigators independently, and then used to guide the indexing of the remaining transcripts. The coding process enabled the development of sub-themes to be charted and organised into overarching themes that manifest within the whole dataset. Once all transcripts were fully coded and checked, the investigator compared the common recurring viewpoints of healthy adults, adults at risk of NCDs, and community-based primary healthcare professionals in order to identify similarities and differences among the three groups.

## Results

A total of 77 persons were invited for the study, of whom 56 adhered to the study protocol and provided sufficient PA data. Of them, 35 participated in interviews. There were no significant differences between interviewed and non-interviewed participants in terms of socio-demographics and anthropometry.

The sample included 18 apparently healthy adults (Mean age 36.7 ± 10.2 years; male 37.6 ± 12.1; female 35.4 ± 6.6), 11 adults at risk of NCDs (Mean age 43.8 ± 7.7 years; male 40.8 ± 8.5; female 45.6 ± 7.3) and 6 primary healthcare professionals (Mean age 42.7 ± 7.6 years; male 41.5 ± 2.1; female 43.3 ± 9.6). All other socio-demographic and anthropometric characteristics of the participants are summarised in Table 1.

**Table 1:** Basic characteristics of the study participants

Characteristics	Adults at risk of NCD (***n*** = 11)	Apparently healthy adults (***n*** = 18)	Healthcare providers (***n*** = 6)
**Sex**			
Male	4 (36.4%)	11 (61.1%)	2 (33.3%)
Female	7 (63.6%)	7 (38.9%)	4 (66.7%)
**Current marital status**			
Single	2 (18.2%)	4 (22.2%)	0 (0%)
Married	9 (81.8%)	14 (77.8%)	6 (100%)
**Highest level of education**			
Grade 1–10	3 (27.3%)	3 (16.7%)	0 (0%)
Passed G.C.E. (O/Level)^a^	0 (0%)	7 (38.9%)	0 (0%)
Passed G.C.E. (A/Level)^a^	3 (27.3%)	3 (16.7%)	0 (0%)
Vocational training	0 (0%)	1 (5.6%)	5 (83.3%)
University education	5 (45.5%)	4 (22.2%)	1 (16.7%)
**Monthly family income (in rupees)**			
> 60,000	7 (63.6%)	5 (27.8%)	6 (100%)
41,000 to 60,000	0 (0%)	4 (22.2%)	0 (0%)
21,000 to 40,000	4 (36.4%)	8 (44.4%)	0 (0%)
≤ 20,000	0 (0%)	1 (5.6%)	0 (0%)
**Current employment status**			
Employed	10 (90.9%)	13 (72.3%)	6 (100%)
Self-employed	1 (9.1%)	2 (11.1%)	0 (0%)
Housewife	0 (0%)	3 (16.7%)	0 (0%)
**Current smoking status**			
Smokers	1 (9.1%)	3 (16.7%)	1 (16.7%)
Non-smokers	10 (90.9%)	15 (83.3%)	5 (83.3%)
**Body Mass Index**			
Normal weight	0 (0%)	7 (38.9%)	3 (50.0%)
Overweight	3 (27.3%)	9 (50.0%)	1 (16.7%)
Obese	8 (72.7%)	2 (11.1%)	2 (33.3%)
**Perceived level of physical activity**			
Sufficiently active	7 (63.6%)	13 (72.2%)	5 (83.3%)
Not sufficiently active	1 (9.1%)	1 (5.6%)	0 (0%)
Don’t know	3 (27.3%)	4 (22.2%)	1 (16.7%)

^a^*GCE* General Certificate of Education, *O/L* Ordinary Level, *A/L* Advanced Level

Four themes were developed after analysing all 35 transcripts; understanding the feedback received on PA profile; perceived novelty of the feedback; motivation to change behaviour; and consideration of the multidimensional nature of PA. To provide an indication of the degree to which experiences that participants voiced were shared across and within populations, the sub-themes identified in transcripts were quantified by means of percentage of respondents who shared that particular view in each group of adults at risk of chronic NCDs, apparently healthy adults and primary healthcare professionals.

### Theme 1 – understanding the feedback received on PA profile (Table 2)

At the first appearance, around 70% of the apparently healthy adults, more than 50% of those at risk of chronic NCDs and a few primary healthcare professionals found some of the graphs and concepts presented in the feedback difficult to interpret. For instance, the colours used in traffic light system, health wheel and spikes to represent different intensities in the activity graphs, as well as the concepts on non-sedentary time, moderate & vigorous bouts, and calorie burn were confusing to some. As a result, most participants required extra help to understand some of the information depicted in the feedback.

**Table 2:** Subthemes and example quotes to support Theme 1

Certain graphics/concepts were confusing	*“My understanding is that this should be a dark blue line if I had worked hard* [wrong interpretation of the colour code]. *I am someone who actually works hard, and this feedback has confirmed it.”* (A female in her 20’s among the primary healthcare professionals)
Need initial help to understand the feedback	*“I understood the activity wheel and its overall interpretation, but I need some help with this section on activities given on a daily basis…It is a little bit complicated to understand it at once.”* (A male in his 30’s among apparently healthy adults)
Understand where own PA does not reach PA guidelines	*“I feel that the amount of physical activities I have been doing is not enough. The graphic presentation helped me to realise this.”* (A male in his 20’s among the apparently healthy adults)
Recognize the link between PA & health	*“Actually, this feedback helped me professionally, it has made me understand the link between health and physical activity more than ever before.”* (A female teacher in her 40’s among those at risk of chronic NCDs)
Believability in data presented	*“On this day, I roughly set the alarm at 5 am, snoozed it at 5.05 am and woke up at 5.10 am. This chart indicates the time I woke up, also the time period I was travelling in the vehicle and the period I was sleeping. It is 100% accurate!”* (A male in his 20’s among the apparently healthy adults)
Preference for graphics above statistics	*“It is better to use pictures rather than numbers because anyone can understand it regardless of their level of education.”* (A female in her 40’s among the adults at risk of chronic NCDs)

After receiving some support in interpreting feedback, participants in all groups found the information believable, which was enhanced by seeing the accuracy with which the timeline reflected the changes in their daily activity. Around half of the non-healthcare participants indicated that understanding visual images given in the feedback was simpler than numbers or statistics, so that complex concepts related to PA could be easily communicated. In comparison, this fact was not raised or identified by the primary healthcare professionals.

### Theme 2 – perceived novelty of the feedback (Table 3)

Wearing the activity monitor and having access to unique features/functionality of the web-platform were perceived as novel features. Most participants had not seen feedback on daily PA presented in this format, which also added to the novelty. Most of the non-healthcare adults thought the feedback provided was ‘unexpected’, thus making them curious to find out more, while all primary healthcare professionals seemed to encounter at least one important fact about their own behaviour that they were previously unaware of. The feedback showed some potential to counteract common social myths about PA, such as whether age is a barrier to PA, that activities of daily living can contribute to fitness and calorie burn, and that even short bouts of exercise can be important. Also, the feedback was novel as it provided new knowledge about PA guidelines as well as awareness on their own behaviour. In contrast, only one adult at risk of NCDs and two apparently healthy adults showed apathy towards the feedback in the day by day activity section, partly because, although such information was novel, it did not provide a straightforward message on the prediction of their disease risk related to PA. This was also coupled to the fact that activity graphs in the day-by-day activity section were more difficult to understand.

**Table 3:** Subthemes and example quotes to support Theme 2

Features are ‘novel and surprising’	*“I was excited since I have never seen a device like this before. Also, I never thought such a small wrist band could provide this much information. It’s very useful and is actually good work.”* (A male in his 20’s among the apparently healthy adults) *“We have never come across this type of feedback in our life. We have seen information like this only twice before. One to indicate the strength of earthquakes, and the other to measure heart rhythm.” (*A male in his 30’s *among the* apparently healthy adults*)*
A mismatch with their initial perception of behaviour	*“No. It’s different than I expected. Though I had assumed I was working hard, such as carrying weights, standing for a long time… I have not.”* (A male in his 20’s among the apparently healthy adults)
Reveals some ‘unexpected’ information	*“This feedback also shows the light activities that I have done. I did not know that we burn this much of calories by doing light activities. I thought these activities are not worthy in terms of calorie expenditure, so I didn’t see any value in these for improving health.”* (A female in her 30’s among the adults at risk of chronic NCDs)
Feedback challenges myths about PA	*“To date, I have thought that we should sit, sleep and rest as much as possible. I thought it is not good to be standing for long hours, because it burns a lot of calories. Today only I got to know that it is actually good, but not good to be sedentary… I can now balance both aspects.”* (A male in his 20’s among the apparently healthy adults)
Increases knowledge of PA	*“Yes, I gained knowledge from this. It increased my knowledge from 1 to 90%.”* (A male in his 20’s among the apparently healthy adults)
Generates apathy towards the feedback provided	*“To the majority, it can be explained by this* [referring to the activity wheel], *and those who can grasp the message would do so quickly. Personally, I don’t like to listen to many things, it can become boring. If it can be quickly explained including how to attain the targets, that should be sufficient.”* (A male in his 50’s among the apparently healthy adults)

### Theme 3 – motivation to change behaviour (Table 4)

Initially on seeing the data, the majority of adults at risk of chronic NCDs, a small proportion of apparently healthy adults and a few primary healthcare professionals appeared to use denial as an immediate coping mechanism, stating that the data presented in the visual feedback is not a typical representation of their normal activity pattern. However, as the discussions progressed, most began to be more open to what the information they received implied. By the end of the interviews, the majority of participants (from all groups) reported being motivated to consider changing their behaviour. The most commonly cited motive for change was the feedback showing large discrepancies between the amount of PA achieved versus the targets recommended for each activity dimension and sedentary time. Thus, features such as “current activity versus guide bars”, “traffic light system” were cited as encouragement for behavioural change by most of the respondents in each study group. While some adults at risk of chronic NCDs considered the targets recommended by the visual feedback to be feasible, only a small number among the apparently healthy adults and primary healthcare professionals showed confidence in achieving them. Almost half of participants spontaneously suggested incorporating more PA within their daily activities to address the deficient calorie burn highlighted in the PA profile in general terms, but when promoted, only a small proportion of the sample were willing to set a specific action plan for changing their behaviour.

**Table 4:** Subthemes and example quotes to support Theme 3

Use denial as an immediate coping mechanism	*“The week I spent wearing this band was different from my usual week owing to the prevailing weather. I had to spend this week with less active work. I couldn’t do any outdoor activities in the last few days. Hence, my level of activity is reported as quite low during the testing period.”* (A male in his 50’s among the apparently healthy adults)
Feedback is motivating	*“When I see this, I think it is an incentive to achieve my goal. With this knowledge, I can bring down my weight and I am now motivated to change my routine.”* (A female in her 40’s among the apparently healthy adults)
Discrepancies from PA targets effective	*“It is good so that we can motivate ourselves to achieve the maximum. We can see the level we have achieved and the level we need to achieve.”* (A male in his 40’s among the primary healthcare professionals)
PA targets are realistic	*“This profile is generated based on my activities of the whole week; comparing this with the activities I did in a day, I think I can do it. I mean, I can achieve this easily, without being too tiered. I think I can change my behaviour to achieve these targets.”* (A male in his 20’s among the apparently healthy adults)
Suggested of incorporating daily activities instead of a proper plan to address the deficient calorie burn	*“I usually climb stairs only once in the morning. At the same time, I bring all necessary things and climb up there at night to sleep only (laughs). Climbing stairs is an activity which I tried to avoid as much as possible. But after seeing this profile, it is also a very useful activity to burn some calories. So, I should do more of that from now on.*” (A female in her 30’s among the adults at risk of chronic NCDs)
Set a plan of action to increase PA	*“I plan to engage in some sort of activities during leisure time.... at least walk for 20 min or do something I can do.”* (A male in his 20’s among the apparently healthy adults)

### Theme 4 – consideration of the multidimensional nature of physical activity (Table 5)

Multidimensional feedback was useful to demonstrate the potential options available for behavioural change and, following feedback, most participants understood that PA is composed of several dimensions that are independently important for health. Around half of the participants appeared to re-evaluate their own current PA levels; and suggest more suitable or tailored options to change behaviour that would fit with the practical issues, physical abilities, and health conditions that they faced. That is, they appeared to find the multidimensional feedback useful in identifying a personally acceptable and feasible response to the feedback. Nonetheless, there was a tendency to focus mainly on calorie burn, above other dimensions, which seemed to be very compelling. This linked to participants’ interest in monitoring their diet as well as PA as a result of the study. There were differences in opinion as to the importance of diet relative to PA, as while some believed that diet is superior to PA for weight loss, the apparently healthy adults in particular were less interested in considering dietary change to balance energy expenditure.

**Table 5:** Subthemes and example quotes to support Theme 4

Identified more suitable options to increase PA	*“As a human, we don’t have to go to the gym for burning energy. We can do it through our daily activities…….A study like this can put a person, who can’t go to a gym properly or who can’t go to a playground, to the right path. Through this platform, how he can make use of his daily activities to improve his health is shown”* (A male in his 50’s among the apparently healthy adults) *“I realized that rather than doing heavy activities, it is better to do a lot of light activities. It is much better to increase the activity time through light intensity activities even in short bouts, rather than struggling to do strenuous activities and trying to satisfy the calorie burning requirement at once.”* (A female in her 30’s among the adults at risk of chronic NCDs)
Mainly focus on calorie burn	“*If we reach the recommended total calories burn, do we have to worry about moderate & vigorous bouts*?” (A female in her 40’s among the primary healthcare professionals)
Coupled with diet/calorie intake	*If I can burn this much of calories by doing these as well as control my eating habits, I might be able to lose about 1 kg a week (laughs).”* (A female in her 30’s among the adults at risk of chronic NCDs)

## Discussion

This paper reports on the feasibility of a digital system that generates multidimensional visual PA feedback to increase understanding of the personal activity level and motivation to increase activity in Sri Lankan adults. This is the first study of its kind in the Asian region.

We explored acceptability and feasibility among three groups: healthcare professionals, adults at risk of NCDs, and adults with no identified health risks. Overall, the experiences of the three groups were largely comparable. For the majority of participants, personal multidimensional feedback was perceived to be informative, acceptable, and sufficiently novel to be relevant and of interest to them. However, some guidance as to how to interpret the various forms of graphical feedback provided was needed, following which the participants were able to identify both their PA level and specific areas where their activity levels were further from recommended amounts. The feedback was considered novel in counteracting common myths about PA mostly among the apparently healthy adults, and in generating interest for behavioural change in almost all participants. Information provided in relation to the number of calories burnt and time spent sedentary was particularly motivating. All three groups had the ability to identify the PA options most suitable for them based on the personalised feedback, however only a small proportion showed confidence and progressed to make firm action plans.

### Understanding and acceptability of the feedback

Some of the themes identified among healthcare professionals, patients and healthy adults in previous studies, such as the ability to enhance knowledge through visual feedback and to understand their own PA profile [13, 23, 24], were also observed in our study, and implies that such feedback is useful for personal reflection and as a source of knowledge among Sri Lankan adults. However, a substantial proportion of adults in each study group needed some support and guidance in interpreting feedback. In contrast, previous research identified a large difference between healthcare professionals and patients from developed countries (80% vs. 28%) on the need for additional support [13]. One reason for this discrepancy between studies could be that, although most adults in Sri Lanka are aware of the importance of being physically active, there is less experience in self-monitoring of PA, even among healthcare professionals. Further, there is lack of consensus on the PA guidance received through experts and mass media, thus Sri Lankan adults receive less and somewhat conflicting information with regards to the type, duration, timing and frequency of PA necessary to promote good health [21, 25]. This suggests that if this intervention is to be rolled out, primary healthcare professionals should be first trained on self-monitoring of PA, and such training be aligned with their ability to convey locally relevant and scientifically valid advice.

With some guidance, the majority of participants across all groups were able to understand their PA profile. Specific features in the feedback that enabled such understanding were its ability to relate to the behaviour in a meaningful manner and having graphical presentations rather than numbers, both demonstrating the acceptability of multidimensional feedbacks in Sri Lanka. In particular, the traffic light system used to represent the achievements in different PA dimensions appeared to be easily understood among other graphics. In the UK, only a very few participants felt that the images were not sufficiently detailed [13], and this was also the case in the current study. However, the notion that visual images are simpler than numbers or statistics was supported only by a few healthcare professionals in the present study, which contrasts with previous research where almost all healthcare professionals and patients in the UK championed this approach as a route to improve the clarity of feedback [13].

### Motivational drive

Prior research has demonstrated the positive motivational properties of personalised feedback [13, 24]. The UK study showed that 83% of patients and 73% of healthcare professionals found the feedback to be inspirational [13]. The corresponding values in our study were remarkably similar at 82 and 67% (in patients and healthcare professionals, respectively), and 72% among healthy adults. Thus, the motivational aspects of the feedback were highly consistent across countries and population groups.

Nearly 70% of the adults in each group of our study cited the discrepancy demonstrated between the actual PA level and health targets as effective for inspiring them to set goals or identify areas for improvement (i.e. current activity vs. guide and traffic light visualisations). Thus, the participants clearly found the feedback to be believable and relevant, which is an important first step towards helping people to initiate and maintain behaviour change.

A unique feature of the feedback was its perceived novelty. A greater proportion of the healthy and unhealthy adults and all healthcare professionals indicated novelty in the type of information provided as well as the way it was presented, so that many aspects of personalised PA profiles were found to be ‘unexpected’ relative to their initial perception of their behaviour. Novelty is recognised as an important component of effective health messaging, as it encourages participants to engage with the information [26, 27]. In the present study, the degree of engagement with the information appeared to be sufficiently strong for participants to challenge some of their own beliefs, including common social myths about PA, such as believing that only intensive activities (e.g. brisk walking and workouts) are essential for improving the overall PA level. However, certain terminology used (e.g. non-sedentary time, vigorous bouts) and colours used to represent the intensity of activities were confusing to the majority in both non-healthcare professional groups, which could be due to some visualizations not being intuitive enough especially for the participants to read and reinterpret. On top of that, such graphics (e.g. activity graphs) provided very detailed information (e.g. minute by minute activities, intensities and amount of calorie burn), yet failed to provide straight-forward information about the potential diseases risk, which was expected by the participants. Incoherence has been reported as a cause of apathy towards some graphics in previous research [16, 28].

### Intention to change and planning

In the present study, 46% of adults at risk of NCDs, 39% of healthy adults and 33% of primary healthcare professionals proposed an action plan to improve PA. These findings were compatible with previous studies on personalized mobile displays [29]. Encouragingly, nearly half of the at-risk adults formed an intention to increase their PA behaviour following a relatively low-cost and simple approach as in our study, indicating the potential cost/benefit value of self-monitoring and feedback for promoting PA in primary care. All healthcare professionals and a majority of healthy adults had at least one ‘amber’ (near guide) coloured dimension in their health profile, whom would have received a message of ‘you are not doing well, but you are not doing terribly badly, either’ (data shown as additional file 2). This self-satisfaction coupled with absence of disease risk could influence the potential need for an action plan to improve PA [14]. However, it should be noted, as shown in literature [30], that translation of intention to change into behaviour is complex and dependent on many other inter-related factors pertaining to PA action control.

### Readiness of primary healthcare professionals

Acceptance among healthcare professionals is extremely important for maintenance of novel interventions or use of health technology. Promisingly, none of the primary healthcare professionals in our sample showed apathy towards the information provided in the visual feedback. Also, a majority of healthcare professionals believed that the visual feedback improves their knowledge related to PA, in contrast to the study from UK, where a smaller proportion of healthcare professionals (20%) acknowledged that the feedback provides new information [13]. This could be, unlike Western countries, Asian region including Sri Lanka is less exposed to technology enabled PA feedbacks across all socio-demographic strata, owing to reduced availability of devices at affordable prices.

With regards to intention to change behaviour, evidence from a recent study from the UK has shown that having received personalised feedback, almost all low active healthcare professionals rationally acknowledged and identified the need to do more PA, irrespective of their earlier views on the level and importance of PA [14]. In comparison, all primary healthcare professionals in the present study too recognised their weaknesses in behaviour and believed that the feedback and self-monitoring could be used to facilitate improvements in PA behaviour, implying their readiness to change.

The overall findings of the study suggest that the technology enabled feedback has key applications at individual level, however the devices should be further explored on their purposive use in terms of specific times (e.g. post-rehabilitation, post-recovery) and context (e.g. insufficiently active adults, sedentary workforce). It should also be noted, that unless these efforts are properly coordinated with other correlates and determinants identified on PA [31], it would not produce a substantial effect on individuals at high risk for physical inactivity.

### Strengths and limitations

The sample required for the qualitative study was compatible with the number recruited (*n* = 35) for a study with similar objectives [13]. Further, the sample was obtained with representation of both low- and high-income populations, thus ensuring the validity of study findings. However, this study was limited to an urban setting, so the findings cannot be generalised to the whole population but only to urban dwellers in Sri Lanka. The interview transcripts were translated to English language, during which the meaning could be affected to a minor degree. However, this translation was undertaken by the investigator who performed the interviews and thus this potential for the original meaning and context to be lost is only likely to be small.

### Conclusions and recommendations

Apparently healthy adults, primary healthcare professionals and adults at risk of NCDs were able to understand technology-enabled personalised multidimensional visual feedback after some guidance. The novelty and unique features of this feedback meant that people found the feedback motivating, and they could use this information to identify areas for improvement and suggest alternative options to increase their PA. However, the feedback (alone) did not trigger the need for a plan of action in the majority and additional support would be required to achieve this goal.

## Supplementary Information

**Additional file 1.** Topic guide and provisional interview schedule - Topic guide includes three main parts. Part 1: interpretation of physical activity profile; Part 2: acceptability of the technology enabled physical activity profiling; Part 3: relevance of technology enabled profiling to patients.

**Additional file 2.** Traffic-light representation of physical activity profiles of participants shown across multiple specific dimensions of physical activity. Green/red indicates achievement/failure to achieve each threshold, while amber indicates that values are near to achieving the threshold.

## Data Availability

All data supporting the findings of this study are contained within the manuscript. Additional information on the study will be shared by the corresponding author on reasonable request.

## Abbreviations

PA: Physical activity
NHS: National Health Service
NCD: Non-communicable diseases
VPA: Vigorous physical activity
MPA: Moderate physical activity
MVPA: Moderate-vigorous physical activities
