# Supplementary material for: The understanding, acceptability, and relevance of personalised multidimensional physical activity feedback among urban adults: evidence from a qualitative feasibility study in Sri Lanka
Source: BMC Public Health. 2021 Apr 13;21:715. doi: 10.1186/s12889-021-10774-0 (PMC8045206; doi:10.1186/s12889-021-10774-0)
Supplement: Supplementary file 1 — Additional file 1. Topic guide and provisional interview schedule - Topic guide includes three main parts. Part 1: interpretation of physical activity profile; Part 2: acceptability of the technology enabled physical activity profiling; Part 3: relevance of technology enabled profiling to patients. [file 12889_2021_10774_MOESM1_ESM.docx]

**Confidential**

**TOPIC GUIDE AND PROVISIONAL INTERVIEW SCHEDULE**

**Research title: Interpretation of technology enabled visual feedback, acceptability and relevance of personalised multi-dimensional physical activity profiling among apparently healthy adults, those at risk of chronic non-communicable diseases and community-based healthcare providers in Pitakotte MOH area**

**PART 1: INTERPRETATION OF PHYSICAL ACTIVITY PROFILE**

**1.1 Perceived physical activity profile**

[Objective: To gauge participants’ understanding of physical activity, as perceived by them]

1.1.1 What sort of activities do you think of, when you talk about physical activity?

*Prompts:*

- Can you give me any examples of things that are physical activity versus things that aren’t?

1.1.2 Do you regularly take part in any physical activity?

*Prompts:*

- If yes, what sort of physical activity do you do?
- If yes, is there anything that helps you to keep active?
- If not, are there any types of physical activity that you do from time to time?
- What are your reasons for taking physical activity if/when you do?
- If not, why do you prefer not to do physical activity?

1.1.3 Would you say your level of activity is typical compared with the people around you?

*Prompts:*

- What do your friends and family think about physical activity?
- Do you ever do physical activity or exercise with other people? If so, who?
- Does what your friends or family think or say make a difference to how active, or inactive you are?

1.1.4 What do you think about the amount of physical activity you do?

*Prompts:*

- Is it too little, too much…. just enough?
- Do you notice any benefits when you are more active?
- What are the benefits to you of not being physically active?
- If you would like to do more, what stops you?

**1.2 Physical activity profile based on the technology enabled visual feedback**

[Objective: Whether the information being presented is clear and useful and how the profile makes the user feel about their physical activity levels etc.

Welcome the participant back, and explain that you will talk through the information that you can download from their monitor, and will discuss what they think of it. Remind them of the reason for this study being to explore if this could be something useful to help people to become more active in future, so there are no right/wrong answers, and any feedback they can give on how they understand the feedback or what is less easy to understand, is important].

*>>Go to HEALTH: The Health Profile section of the website.*

1.2.1 What do you think of this feedback?

*Prompts:*

- Do you understand what it is showing you?
- How does seeing your physical activity data in this way make you feel?

*>>Go to ACTIVITY: The Activity section of the website*

1.2.2 What do you think of this feedback?

*Prompts:*

- Do you understand what it is showing you?
- How does seeing your physical activity data in this way make you feel?

*>>Go to TAGGING, then PLANNING: The Tagging and Planning sections of the website*

1.2.3 What do you think of these features of the website?

Prompts:

- Do you understand what it is showing you?
- How does seeing your physical activity data in this way make you feel?

>> Reflecting on all aspects of the feedback…

1.2.4 Is any of this surprising?

*Prompts:*

- Were the energy costs as you expected for the activities you did?
- Did your personal profile provide you with information you were not aware of? If so, what?

1.2.5 Did seeing your feedback like this give you any better understanding of your own physical activity and how it links to your health?

*Prompts:*

- If yes, what part was most useful?
- If yes, what did you learn?
- If no, can you tell us more about why not?

**PART 2: ACCEPTABILITY OF THE TECHNOLOGY ENABLED PHYSICAL ACTIVITY PROFILING**

[Objective: What users do and don’t like about their personal profile options (colours, detail, text, etc.)]

2.1 Would you feel comfortable wearing the wristband to monitor your physical activity in future?

*Prompts:*

- If yes, why?
- If not, why?

2.2 What features of the [individual] design do you like? Why?

2.3 Which bits do you not like? Why?

2.4 Is there anything we haven’t talked about that you would like to say about your experience wearing the devices, or looking through you feedback?

**PART 3: RELEVANCE OF TECHNOLOGY ENABLED PROFILING TO PATIENTS**

[Objectives: To gather whether the personal profile have perceived educational and motivational properties, and whether the users see value in progress tracking, goal setting, and progressive detail]

3.1 What do you know of the link between physical activity and health?

*Prompts:*

- Do you think physical activity is important for your own health?
- Could it improve your health if you did more?

3.2 Does seeing your physical activity feedback in this way motivate you to want to make any changes?

*Prompts:*

- If yes, in what way?
- If no, what would you need to see for you to feel the need to change?

3.3 If you were going to make a change to your activity level following this feedback, what would you do first?

*Prompts:*

- Do you feel confident that you could make this change?
- What support do you think would help you to make the change?
- Would you find it helpful to have on-going feedback like you have seen today?
- How would you want to get that feedback – in person, on your own device etc.?

3.4 Does your personal profile make you think that you have several options to increase your physical activity?

3.5 Would you consider using the profile to monitor your behaviour? How?

3.6 Do you think that your personal profile provided you with information you were not aware of? If yes, what was that information? Do you think that technological platforms to display and track profiles could be beneficial? Do you think they could provide practical information?

[These are additional questions to be asked only from healthcare providers. Before starting on Part 3, explain to the health care provider that the following questions are about how his/her patients would respond to profiling.]

3.7 Thinking about the patients you work with, what do you think they would make of this feedback?

*Prompts:*

- Do you think they would be willing to wear the devices?
- Do you think this feedback would be interesting and relevant to them?

3.8 Can you see this being feasible for you to use with your patients day to day?

*Prompts:*

- Would you feel confident in providing feedback like I have done today?
- What might get in the way of you using this?
- What would encourage you to use this?

3.9 Is there anything we haven’t talked about that you would like to say about your experience wearing the devices, or how you think they would work with patients?

**CLOSE**

Thank you very much for taking the time to participate. Please feel free to ask any questions about anything we have discussed.
