# Supplementary figures and images for: The understanding, acceptability, and relevance of personalised multidimensional physical activity feedback among urban adults: evidence from a qualitative feasibility study in Sri Lanka
Source: BMC Public Health. 2021 Apr 13;21:715. doi: 10.1186/s12889-021-10774-0 (PMC8045206; doi:10.1186/s12889-021-10774-0)

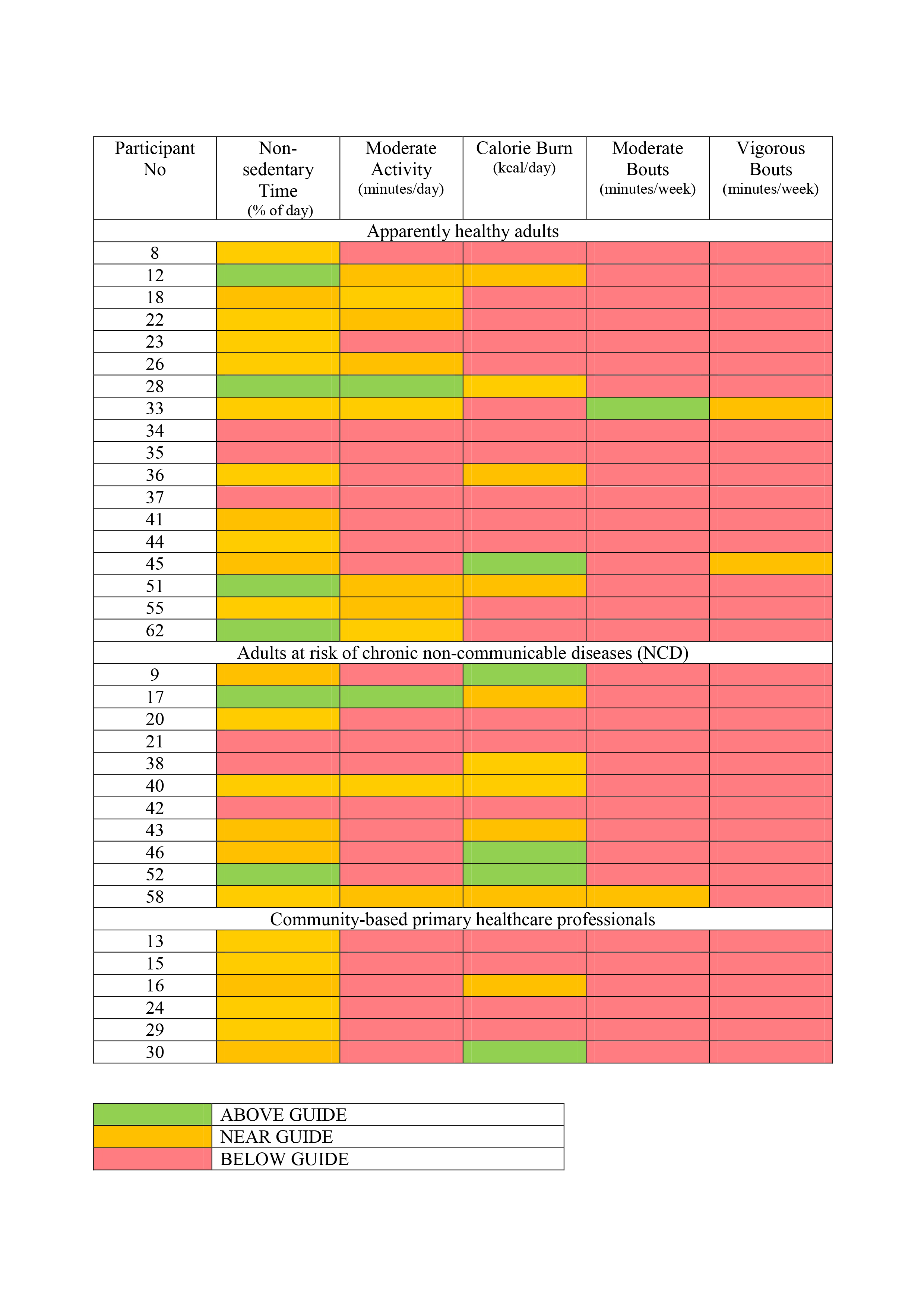

Supplement: Supplementary file 2 — Additional file 2. Traffic-light representation of physical activity profiles of participants shown across multiple specific dimensions of physical activity. Green/red indicates achievement/failure to achieve each threshold, while amber indicates that values are near to achieving the threshold. [file 12889_2021_10774_MOESM2_ESM.jpg]
